# Interplay between circadian and other transcription factors—Implications for cycling transcriptome reprogramming

**DOI:** 10.1002/1873-3468.70232

**Published:** 2025-11-28

**Authors:** Xinyu Y. Nie, Jerome S. Menet

**Affiliations:** ^1^ Department of Biology, Center for Biological Clock Research Texas A&M University College Station TX USA; ^2^ Interdisciplinary Program of Genetics Texas A&M University College Station TX USA

**Keywords:** Circadian clocks, cis‐regulatory elements, CLOCK:BMAL1, nucleosome, reprogramming, single‐molecule footprinting, transcription, transcription factor cooperativity

## Abstract

Circadian transcription factors (TFs) orchestrate daily rhythms in gene expression to drive rhythmic biological functions. In mammals, this system relies on the TF CLOCK:BMAL1, which binds E‐boxes to initiate rhythmic transcription. While traditionally viewed as a master activator, CLOCK:BMAL1 is now recognized to engage in additional regulatory functions that are essential for its activity. This perspective focuses on the mammalian circadian clock and integrates genomic, structural, and single‐molecule footprinting data to highlight emerging insights into how CLOCK:BMAL1 regulates chromatin architecture, cooperates with other TFs, and coordinates complex enhancer dynamics. We propose an updated framework for how circadian TFs operate within dynamic and multifactorial chromatin landscapes, and prime cis‐regulatory elements for rhythmic transcriptional bursts. We also discuss how this framework underlies circadian reprogramming and transcriptional plasticity.

## Graphical abstract

This perspective highlights emerging insights into how the circadian transcription factor CLOCK:BMAL1 regulates chromatin architecture, cooperates with other transcription factors, and coordinates enhancer dynamics. We propose an updated framework for how circadian transcription factors operate within dynamic and multifactorial chromatin landscapes, and prime cis‐regulatory elements for rhythmic transcriptional bursts. We also discuss how this underlies circadian reprogramming and transcriptional plasticity.

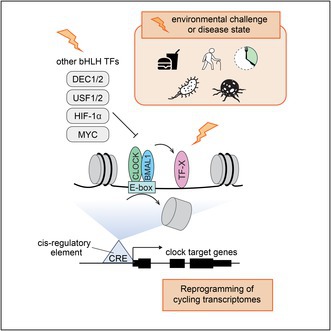

## Abbreviations

**BAF**, BRG/BRM‐associated factor

**bHLH**, basic helix–loop–helix

**CAP‐SELEX**, consecutive affinity‐purification systematic evolution of ligands by exponential enrichment

**CCG**, clock‐controlled gene

**ChIP‐reChIP**, sequential chromatin immunoprecipitation

**Co‐IP**, co‐immunoprecipitation

**CRE**, cis‐regulatory element

**HDAC**, histone deacetylase

**NCoR**, nuclear receptor co‐repressor

**NOMe‐seq**, nucleosome occupancy and methylome sequencing

**NR**, nuclear receptor

**Pol II**, RNA polymerase II

**RORE**, ROR element

**SMF**, single‐molecule footprinting

**TF**, transcription factor

**TTFL**, transcription‐translation feedback loop

## Introduction

The circadian clock is a timekeeping mechanism that enables organisms to anticipate and adapt to the daily variations of their environment. Found across most taxa, the circadian system has independently evolved multiple times reflecting its fundamental importance for organismal fitness and survival [1, 2, 3]. In eukaryotes, circadian rhythms are built upon transcription‐translation feedback loops (TTFLs) that generate self‐sustained oscillations in gene expression with a 24‐h periodicity.

**Fig. 1:**
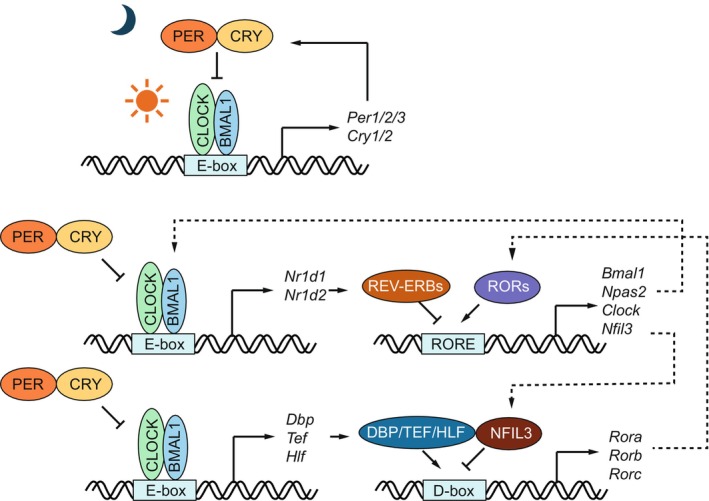
Transcriptional architecture of the mammalian circadian clock. The mammalian circadian clock is organized in transcription‐translation feedback loops initiated by the transcription factor CLOCK:BMAL1, which actives the rhythmic transcription of the repressors PER and CRY. CLOCK:BMAL1 also regulates the rhythmic expression of additional transcriptional regulators that form interconnected secondary loops, including the nuclear receptors RORα/β/γ and REV‐ERBα/β, as well as the PAR bZIP transcription activators DBP, TEF and HLF, and repressor NFIL3. Solid arrows indicate gene transcription and protein translation. Dotted arrows are used to indicate the interconnected secondary loops. Blunt arrows indicate inhibition.

In mammals, the core TTFL relies on the basic helix–loop–helix (bHLH) transcription factors (TFs) BMAL1 and CLOCK, or its functionally redundant paralog NPAS2. These proteins form CLOCK:BMAL1 or NPAS2:BMAL1 heterodimers, which bind DNA on E‐box elements during the day to activate the transcription of *Period* (*Per1/2/3*) and *Cryptochrome* (*Cry1/2*) genes (Fig. 1) [4, 5, 6]. The PER and CRY proteins accumulate in the cytoplasm, dimerize, and translocate to the nucleus at night, where they repress CLOCK:BMAL1 (and NPAS2:BMAL1) activity, thereby inhibiting their own transcription. As PER and CRY proteins are progressively degraded, repression is relieved, and a new cycle begins. Beyond this primary feedback loop, CLOCK:BMAL1 also regulates the rhythmic expression of additional transcriptional regulators that form interconnected secondary loops. They include the nuclear receptors RORα/β/γ and REV‐ERBα/β (also known as NR1D1/2), which rhythmically bind ROR elements (ROREs) to respectively activate and repress the transcription of *Bmal1*, *Clock*, *Nfil3*, and other target genes. Additionally, the PAR bZIP transcription activators DBP, TEF, and HLF, along with the PAR bZIP transcription repressor NFIL3 (also known as E4BP4) bind D‐box elements to regulate the expression of *Rev‐erb* and *Ror* genes, as well as clock output genes (Fig. 1). These interlocked regulatory loops enhance the robustness and temporal precision of circadian transcriptional programs, and account for the diverse yet fine‐tuned phases of rhythmic gene expression [7, 8].

Although NPAS2:BMAL1 is often viewed as functionally redundant with CLOCK:BMAL1, its precise contribution remains less clearly defined. NPAS2 binds roughly half as many genomic sites as CLOCK, with ~ 76% of NPAS2 peaks overlapping those of CLOCK, suggesting that CLOCK:BMAL1 is the predominant driver of circadian transcription, and that NPAS2 plays a more limited, overlapping role [9]. Nonetheless, it remains to be determined whether NPAS2:BMAL1 confers distinct chromatin interactions or tissue‐specific functions beyond its redundancy with CLOCK:BMAL1, and whether the principles discussed below for CLOCK:BMAL1 extend to NPAS2:BMAL1.

Although the TTFL model positions CLOCK:BMAL1 as a key driver of rhythmic transcription, genome‐wide studies have revealed a more complex picture. Notably, a substantial fraction of the genes directly targeted by CLOCK:BMAL1 are not rhythmically expressed [10, 11, 12]. This finding, along with other reports (see below), challenges the notion that CLOCK:BMAL1 rhythmic DNA binding automatically translates to rhythmic transcription. They also suggest that additional layers of regulation, which include the involvement of other noncircadian TFs, are essential for circadian gene output.

In this perspective, we examine recent findings that highlight how CLOCK:BMAL1 reshapes the chromatin landscape to facilitate context‐dependent interactions with other TFs. These cooperative and competitive interactions provide a molecular framework to explain how cycling transcriptomes are fine‐tuned at the level of individual enhancers, how they integrate environmental cues, and how they become reprogrammed in response to physiological and pathological states. By focusing on the chromatin‐level mechanisms of CLOCK:BMAL1 function and the interplay between CLOCK :BMAL1 and other TFs, we aim to highlight emerging principles of enhancer regulation, TF cooperativity, and circadian plasticity.

## Transcription of CLOCK:BMAL1 targets largely relies on CLOCK:BMAL1 cooperation with other transcription factors

Circadian clocks are essential for pervasive and coordinated rhythms in gene expression. In mammals, about half of all protein‐coding genes exhibit rhythmic expression in at least one tissue, with each organ displaying a unique circadian transcriptome shaped by its physiological functions [13, 14, 15, 16]. Early studies investigating the underlying mechanisms revealed that the transcription of many clock‐controlled genes (CCGs) depends on additional regulatory layers than just circadian TFs (used to encompass TFs that make up the TTFLs). In particular, while rhythmic binding of circadian TFs to DNA was necessary for rhythmic transcription, it was not by itself sufficient to generate overt rhythms [11].

This conclusion is supported by the striking disconnection between CLOCK:BMAL1 chromatin occupancy and the expression of its target genes. Genome‐wide studies show that CLOCK:BMAL1 DNA binding is rhythmic and peaks during the day [9, 17], yet the majority of its target genes are not rhythmically transcribed [11, 12]. Epigenomic data further reveal that the activity of cis‐regulatory elements (CREs; encompassing enhancers and promoters) bound by CLOCK:BMAL1 correlates poorly with CLOCK:BMAL1 daytime binding, but instead aligns with the transcriptional output of target genes. For instance, marks associated with active CRE, such as H3K27ac enrichment, enhancer RNA production, and RNA polymerase II (Pol II) recruitment, are rhythmic at CLOCK:BMAL1‐bound CREs when the associated target genes are transcribed rhythmically. Conversely, signals from these marks are arrhythmic when target genes are constitutively expressed [12].

Additional evidence also demonstrates that circadian transcriptomes are highly plastic and responsive to environmental cues or disease states, even when TTFL oscillations remain intact. This phenomenon, originally termed ‘*circadian reprogramming*’ by Paolo Sassone‐Corsi and colleagues, has been observed under a wide range of conditions, including fasting, high‐fat diet, aging, and inflammation [18, 19, 20, 21, 22, 23, 24, 25]. Importantly, such reprogramming affects genes directly targeted by CLOCK:BMAL1 with, for instance, some targets gaining rhythmicity specifically under fasting [12]. These observations underscore that environmental signals reshape TF networks that interact with CLOCK:BMAL1, thereby rewiring the landscape of rhythmic gene expression.

Finally, the rhythmic expression of CCGs is critical for the rhythmicity of biological processes, which often occurs in specific tissues. As such, the expression of CCGs is also regulated by master TFs that control biological pathways, such as *Pparα/γ* and *Srebp1/2* for lipogenesis, *NF‐κB* for inflammation, and *Nrf1/2* for mitochondrial function. This strongly implies that circadian transcription arises from the interplay between CLOCK:BMAL1 and these master regulators rather than from circadian TFs alone. Experimental evidence supports this model, for example, activation of NF‐κB by inflammatory stimuli relocalizes CLOCK:BMAL1 genome‐wide to sites bound by NF‐κB, and alters the cycling of inflammatory genes [19]. Additionally, circadian output is to a large extent tissue‐specific [13, 14, 15, 16], indicating that circadian TFs also interact with tissue‐specific TFs. This is supported by data showing that BMAL1 cistromes largely differ between tissues due to CLOCK:BMAL1 cooperation with tissue‐specific TFs [26].

Together, these findings support a model in which CLOCK:BMAL1 transcriptional output requires the cooperation of CLOCK:BMAL1 with other non‐circadian TFs. This model also provides a mechanistic basis for circadian plasticity in response to physiological and environmental changes.

## Experimental approaches to study transcription factor cooperativity—advances and limitations

The investigation of TF cooperativity has long relied on a variety of molecular assays, beginning with classical *in vitro* techniques and evolving toward increasingly sophisticated *in vivo* approaches. Pioneering studies in the 1980s introduced electrophoretic mobility shift assays to detect protein‐DNA complexes based on mobility shifts in native gels [27, 28]. Shortly thereafter, reporter gene assays, including those based on firefly luciferase or chloramphenicol acetyltransferase, were developed to quantify TF‐induced transcriptional activity and TF‐TF cooperativity in cultured cells [29, 30]. The more recent application of CAP‐SELEX (consecutive affinity‐purification systematic evolution of ligands by exponential enrichment) enables high‐throughput, systematic analysis of cooperative DNA binding across thousands of TF pairs [31]. This method uses randomized pools of short DNA fragments to assess binding preferences and combinatorial logic, with a particular focus on short‐range cooperativity and dimer formation. While these *in vitro* approaches provide powerful biochemical insights into TF‐DNA and TF‐TF interactions and/or cooperativity, they lack the chromatin context and spatial complexity that shape transcriptional regulation *in vivo*.

TF cooperativity in the native genome often involves long‐range interactions, higher order protein complexes, and dynamic chromatin environments, which are features that are difficult to recapitulate outside of cells. Moreover, chromatin features such as nucleosome positioning [32, 33], DNA methylation [34, 35, 36], and histone modifications [37, 38, 39] critically influence TF binding, yet are absent in classical *in vitro* systems. To address these limitations, several *in vivo* methods have been developed, each with its own strengths and constraints. For example, ChIP‐reChIP (sequential chromatin immunoprecipitation) allows testing of co‐occupancy between two TFs on the same chromatin fragment but requires large numbers of cells and is technically demanding [40]. Co‐immunoprecipitation (co‐IP) can confirm protein–protein interactions but lacks genomic specificity. Genetic models, including TF knockouts and overexpression systems, provide functional data but are often nonviable for essential genes and lack spatial resolution. CRISPR interference offers scalability and locus specificity but may not fully recapitulate endogenous TF function or dynamic binding behavior [41].

To infer TF occupancy and cooperativity more broadly across the genome, several chromatin accessibility assays have also been leveraged. Techniques such as DNase‐seq, MNase‐seq, and ATAC‐seq, combined with digital footprinting, have enabled inference of TF binding patterns [42, 43, 44, 45, 46]. Each technique, however, comes with trade‐offs. DNase I exhibits cleavage periodicity of about 10 bp on nucleosomal DNA [47], complicating precise footprint mapping. MNase‐seq enables high‐resolution analysis of nucleosome organization, including V‐plots to infer TF‐nucleosome dynamics, but remains a bulk measurement that masks cell‐to‐cell and molecule‐to‐molecule heterogeneity [42, 48]. ATAC‐seq mitigates these shortcomings but lacks definitive evidence for TF cobinding.

To overcome these limitations, DNA methyltransferase‐based single DNA molecule footprint approaches, including single‐molecule footprinting (SMF) and Fiber‐seq, have emerged as powerful *in vivo* techniques for dissecting TF cooperativity in the native chromatin landscape. SMF is based on the labeling of accessible DNA with GpC methyltransferase (M.CviPI), followed by bisulfite sequencing, enabling high‐resolution maps of nucleosome and TF occupancy on individual DNA molecules [49, 50, 51, 52, 53, 54]. The method builds on the foundational work of NOMe‐seq (Nucleosome Occupancy and Methylome Sequencing) [49] but incorporates targeted enrichment for CREs and increased sequencing depth, allowing for base‐resolution analysis of TF binding, nucleosome competition, and chromatin heterogeneity, all while preserving the native epigenomic context. Despite its transformative potential, SMF is not without limitations. The approach depends on the density of GpC dinucleotides (median spacing in mammalian CREs ~ 14 bp [52]), limiting resolution in GpC‐poor regions. Furthermore, bisulfite‐converted DNA presents challenges for downstream applications such as multiplexed targeting or capture. New adaptations including hybridization‐based capture [52] and Cas9/sgRNA‐directed enrichment [55] have expanded genomic coverage, but introduce additional complexities, including off‐target effects, capture bias, and uneven fragmentation.

Fiber‐seq combines labeling exposed adenosines by adenosine methyltransferases with PacBio long‐read sequencing to map TF and nucleosome occupancy along individual DNA molecules with near base‐pair resolution (average adenosine frequency is ~ 1 per 2 bp on double‐stranded DNA) [56]. Thus, it provides a powerful tool to resolve protein footprints on single DNA fibers at high resolution. However, because it relies on direct detection of methylation signals, this approach does not allow for PCR‐based target enrichment, resulting in relatively low coverage at specific loci. Achieving comparable coverage therefore requires substantially higher input material and/or sequencing depth, which poses challenges when working with rare or limited samples.

Nevertheless, SMF and Fiber‐seq represent a conceptual and technical advance in our ability to interrogate TF cooperativity at the level of single DNA molecules, in the presence of native chromatin features. In the context of circadian biology, they offer powerful tools to dissect how CLOCK:BMAL1 interacts with nucleosomes and with other TFs to establish daily rhythms in enhancer activity and gene expression.

## Single‐molecule footprinting reveals the chromatin architecture at cis‐regulatory elements—insights and open questions

Early developments in single‐molecule chromatin footprinting with NOMe‐seq [49] along with the more recent SMF studies demonstrate that M.CviPI‐based SMF is sufficiently sensitive to detect TF footprints *in vivo* in mammalian genomes [48, 52, 53, 54]. Importantly, these studies reveal that SMF can distinguish high‐ vs. low‐affinity binding sites [32], identify cooperative interactions based on motif spacing, and track activation thresholds at CREs. These insights emphasize the utility of SMF for uncovering the rules of enhancer grammar and the interplay between TF binding and chromatin context.

**Fig. 2:**
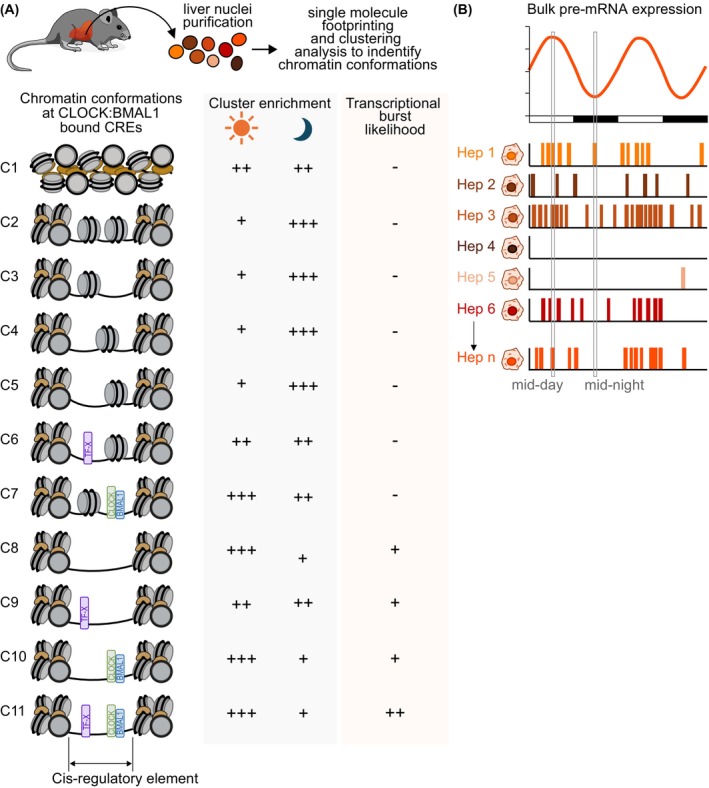
Identification of stereotypic chromatin states at CREs with single‐molecule footprinting and their relationship with transcriptional bursts. (A) SMF enables the detection of multiple chromatin states at cis‐regulatory elements (CREs), each reflecting distinct organization of nucleosome occupancy and transcription factor binding. The relative proportion of these states is dynamic at CLOCK:BMAL1‐bound CREs and changes in a time‐dependent manner, linking chromatin architecture to transcriptional burst likelihood and daily rhythms in gene expression. (B) Model linking transcriptional bursting, its frequency across the 24‐h day at the single‐cell level, and circadian transcription. The peak of expression of a cycling gene is associated with an increase proportion of cells exhibiting transcriptional bursts, and with increased proportion of chromatin states permissive to transcription activation.

Our investigation of the chromatin landscape at CLOCK:BMAL1‐bound CREs in mouse liver using SMF along with a targeted amplicon enrichment strategy achieved consistent, high‐resolution coverage across tens of E‐box‐containing CREs [53]. Through a clustering strategy of DNA molecules based on shared GpC methylation footprints, CREs were found to harbor a limited set of chromatin conformations that represent defined locations and arrangements of nucleosomes and TF binding events (Fig. 2A). These chromatin conformations were remarkably conserved between all samples representing various levels of CLOCK:BMAL1 DNA binding affinity, thereby indicating that chromatin at CREs adopts a small number of structurally constrained stereotypic states.

A striking and unexpected finding was the relative invariance of chromatin states across timepoints and in the absence of CLOCK:BMAL1 (in *Bmal1* knockout mice). This suggests that CRE architecture is remarkably robust, potentially defined by intrinsic DNA features and/or stabilized by TF other than CLOCK:BMAL1. These findings open several key avenues and questions for further investigation:

- What is the functional significance of these discrete chromatin states? Are these states associated with distinct transcriptional outcomes or co‐factor recruitment?
- How dynamic are these states? Do they interconvert rapidly or reflect stable, lineage‐encoded configurations?
- What governs nucleosome positioning at CREs, and how are nucleosomes selectively displaced, remodeled, or stabilized?
- How do chromatin states interface with Pol II loading, enhancer‐promoter communication, and transcriptional bursting?

SMF approaches provide means to explore these fundamental questions. By preserving molecule‐level resolution and chromatin context, SMF offers an unparalleled opportunity to uncover the logic of TF cooperativity and chromatin state transitions *in vivo* in complex regulatory systems like the circadian clock.

### CLOCK:BMAL1‐mediated regulation of chromatin dynamics

Transcription occurs in stochastic bursts, during which multiple Pol II initiation events are interspersed with refractory periods of inactivity [57, 58, 59]. Enhancer strength is a key determinant of burst dynamics, primarily by regulating burst frequency [57, 60]. Our SMF data revealed that chromatin states were remarkably conserved across time points and CLOCK:BMAL1 genotypes. We propose that this conservation of chromatin conformations across conditions and genotypes provides a regulatory scaffold that is all but random. This would allow TFs like CLOCK:BMAL1 to access their motifs in a reproducible manner, such that they can initiate transcriptional bursts in a defined continuous set of active and inactive events (Fig. 2A,B).

While chromatin states were conserved across conditions, their relative abundance varied across time and genotypes. For instance, open states marked by reduced nucleosome footprints were more prevalent at times of strong CLOCK:BMAL1 binding (Fig. 2A). This suggests that CLOCK:BMAL1 does not establish new chromatin configurations, but rather modulates the frequency of pre‐existing chromatin states, particularly those permissive to TF co‐occupancy and transcriptional activation. TF residence time and binding frequency have been directly linked to burst amplitude and frequency, respectively [54, 61]. We propose that CLOCK:BMAL1 promotes rhythmic transcription by redistributing chromatin state probabilities toward TF‐accessible, cooperative configurations, thereby enhancing the frequency of transcriptional bursting. Specifically, the pioneer‐like activity of CLOCK:BMAL1 may contribute to nucleosome destabilization [53, 62], facilitating access for partner TFs. These TFs may differ in their DNA‐binding kinetics, such that their dynamic interactions and co‐occupancy within certain chromatin states eventually reach a cooperative threshold necessary for Pol II engagement and productive transcription. Nevertheless, several key questions remain unresolved:

- How do chromatin states transition dynamically across the circadian cycle?
- Which states are selectively enriched for Pol II, active histone marks like H3K27ac, or histone variants such as H2A.Z, which facilitates nucleosomal DNA unwrapping [63]?
- What mechanisms regulate the proportions of transcriptionally active states in response to circadian time and environmental cues?

Addressing these questions will be essential to understanding how enhancer logic integrates clock timing, chromatin architecture, and TF cooperativity to orchestrate rhythmic gene expression.

### CLOCK:BMAL1 functions as a pioneer‐like transcription factor

In addition to its canonical role as a transcription activator, CLOCK:BMAL1 exhibits features of a pioneer TF, a class of TFs capable of binding nucleosomal DNA and initiating chromatin remodeling to facilitate access for other factors [62, 64, 65, 66]. Support for this pioneer‐like activity comes from MNase‐seq profiling across the 24‐h day. At CREs bound by CLOCK:BMAL1, nucleosome occupancy exhibits a pronounced diurnal rhythm, with lowest occupancy coinciding with maximal CLOCK:BMAL1 binding during the day. These nucleosome rhythms are abolished in *Bmal1* knockout mice, with occupancy remaining uniformly high across time points, indicating that CLOCK:BMAL1 is both necessary and sufficient to mediate rhythmic nucleosome eviction [62]. CLOCK:BMAL1 can bind mononucleosomes in mouse liver, further supporting its ability to engage chromatinized DNA and promote rhythmic nucleosome eviction.

A recent cryo‐electron microscopy study provided structural insights into how CLOCK:BMAL1 interacts with nucleosomes. CLOCK:BMAL1 preferentially binds E‐box motifs located at nucleosome entry–exit sites, with its PAS domain occluding one of the two H2A‐H2B acidic patches. This configuration is incompatible with binding by chromatin remodelers such as the BRG/BRM‐associated factor (BAF) complex, which requires access to both acidic patches [64]. However, CLOCK:BMAL1 may still destabilize nucleosomes by actively recruiting chromatin‐modifying and/or remodeling enzymes through direct protein–protein interaction, thereby remodeling enhancer accessibility in a rhythmic manner.

Leveraging the single‐molecule resolution of SMF, we developed a computational strategy to infer nucleosome protection at individual GpC dinucleotides for each chromatin state [53]. This revealed a subset of nucleosomes harboring E‐box motif(s) at the entry–exit site, which are preferentially targeted by CLOCK:BMAL1 and show decreased occupancy during the day at peak TF binding, suggesting that nucleosome eviction by CLOCK:BMAL1 is highly position‐dependent [53]. These findings align with the structural model and support a mechanism in which CLOCK:BMAL1 selectively engages nucleosomes based on motif topology and spatial constraints.

### CLOCK:BMAL1‐mediated chromatin opening can facilitate the loading of transcription factors

Nucleosomes pose significant steric and structural barriers to TF DNA binding. By wrapping ~147 base pairs of DNA, nucleosomes occlude many TF recognition motifs and impose a rigid DNA conformation that limits both the accessibility and flexibility required for stable TF‐DNA and TF‐TF interactions [33, 67, 68]. For this reason, CLOCK:BMAL1‐mediated chromatin opening and/or nucleosome sliding likely facilitate CRE accessibility to other TFs. This model is supported by the concept of ‘facilitated repression’, in which CLOCK:BMAL1 recruits the SWI/SNF PBAF chromatin remodeling complex to open chromatin and enable REV‐ERBα loading at specific regulatory sites [69]. More broadly, chromatin opening by CLOCK:BMAL1 may create a permissive landscape for cooperative TF binding, including environmentally regulated TFs that otherwise cannot access nucleosomal DNA.

SMF provides an unprecedented resolution to explore this model. First, SMF precisely maps nucleosome occupancy and can quantify at each GpC dinucleotide the proportion of CREs that are nucleosome‐free versus nucleosome‐occupied. As such, SMF can estimate the extent to which DNA is exposed at CREs, and help resolve the complex MNase‐Seq profiles caused by heterogeneous nucleosome positioning [53, 70]. Second, SMF can directly measure TF protection at specific motifs and assess how this protection varies based on chromatin accessibility or with experimental conditions. When paired with clustering analysis and identification of stereotypic chromatin states, SMF can determine whether increased TF occupancy results from improved DNA binding affinity or greater DNA accessibility, simply by comparing TF protection from all reads versus from reads where DNA is exposed at a motif [53].

Application of SMF to CLOCK:BMAL1‐bound CREs reveals that CLOCK:BMAL1 DNA binding is associated at some CREs with increased protection at nearby (> 100 bp) motifs, consistent with cooperative loading of noncircadian TFs [53]. Importantly, this increased protection by non‐circadian TFs does not primarily stem from improved intrinsic binding capability. Rather, the data suggest that it is due to an increased representation of chromatin states where the TF motif is exposed. It is noteworthy that SMF cannot identify the bound TFs nor definitively prove that DNA protection reflects direct protein binding, and complementary antibody‐based assays such as ChIP‐qPCR or ChIP‐seq need to be used to validate and extend these observations.

Notably, SMF also uncovered evidence of long‐range cooperativity between CLOCK:BMAL1 molecules binding to distant E‐box motifs separated by over 250 bp. While short‐range cooperativity at dual E‐boxes separated by 6–7 bp has previously been observed [71, 72] and involves direct protein–protein interaction [64, 71, 73], the physical separation in this case (~ 80 nm) precludes such contacts. Importantly, this cooperativity was only detected at times of high CLOCK:BMAL1 DNA binding, suggesting temporal modulation via time‐specific cofactors and/or post‐translational modifications. This may involve for example the sumoylation of BMAL1 at Lys259, which is rhythmic, occurs in a CLOCK‐dependent manner *in vivo*, coincides with the activation phase of CLOCK:BMAL1, and promotes CBP recruitment to enhancers, thereby enhancing transactivation [74, 75]. *In vitro* assays also showed that BMAL1 phosphorylation, monoubiquitylation, and sumoylation facilitate the recruitment of transcriptional machinery, leading to transcriptional bursting of target genes such as *Dbp* [76]. Independently of the role of time‐specific cofactors and/or post‐translational modifications, this ‘long‐distance’ cooperativity raises the intriguing possibility that CLOCK:BMAL1 may cooperate with noncircadian TFs over similarly long distances, dynamically engaging in enhancer co‐occupancy networks that vary with circadian time or physiological state.

Together, these findings support a model in which CLOCK:BMAL1‐driven chromatin opening promotes DNA accessibility at CREs, thereby enabling opportunistic loading of other TFs regardless of their DNA‐binding capabilities. These TFs do not necessarily bind more efficiently but instead gain access simply because the nucleosomes have been evicted or repositioned. Whether Pol II loading is enhanced when CLOCK:BMAL1 cobinds with other TFs remains to be determined, but this possibility aligns with models in which multifactor binding confers enhancer activation and transcriptional output [54, 57, 60].

## Competition for E‐box access between CLOCK:BMAL1 and other bHLH transcription factors is selective to cis‐regulatory elements

CLOCK:BMAL1 is a member of the bHLH TF family, which encompasses over 100 proteins in mammals, many of which regulate cell‐type‐specific and environmentally responsive transcriptional programs [77, 78]. bHLH TFs generally bind to E‐box motifs (CANNTG), and CLOCK:BMAL1 exhibits preferential binding to the canonical E‐box motif CACGTG, a sequence that is also recognized by several other bHLH proteins, including USF1/2, DEC1/2, and MYC. This motif redundancy inherently creates the potential for widespread competition among bHLH TFs at CREs.

Direct evidence for such competition emerged from studies examining functional antagonism between CLOCK:BMAL1 and other bHLH factors. For example, DEC1/2 and their *Drosophila* ortholog clockwork orange (cwo) act as transcriptional repressors that bind E‐boxes to inhibit CLOCK:BMAL1‐mediated transcription, particularly during the repressive phase of the circadian cycle [79, 80, 81]. Similarly, USF1 competes with CLOCK:BMAL1 for E‐box binding, with enhanced competition observed against the transcriptionally compromised CLOCK^Δ19^ : BMAL1 heterodimer [82]. MYC, another E‐box‐binding bHLH TF, can also antagonize circadian transcription by competing at shared genomic sites [83]. More nuanced interactions have also been described. For instance, CLOCK:BMAL1 can synergistically interact at E‐boxes with the hypoxia‐inducible factor HIF1α and its partner ARNT, and BMAL1 has also been reported to directly form a dimer with HIF1α that binds to CACGTG motifs [84, 85].

While these studies suggest that competition among bHLH TFs is a general principle, ChIP‐seq data reveal limited overlap between CLOCK:BMAL1 peaks and those of other bHLH factors [82, 84]. This indicates that competitive binding is for the most part CRE‐specific and likely influenced by additional contextual factors, including chromatin state, nucleosome positioning, DNA methylation, TF concentration and cofactor availability. This notion is supported by SMF analysis of CLOCK:BMAL1‐bound CREs, which revealed that only a subset of E‐boxes is occupied/protected in BMKO mice [53].

Temporal dynamics also play a crucial role in shaping E‐box competition. The relative abundance, post‐translational modifications, and DNA‐binding competence of bHLH TFs vary across the circadian cycle and in response to environmental stimuli. Thus, the occupancy of shared E‐box motifs likely depends on the relative competition between bHLH TFs over time. In *Drosophila*, cwo preferentially represses CLOCK : CYCLE activity during the subjective night, when CLOCK : CYCLE DNA binding is reduced [81, 86]. In mammals, fluctuations in USF1 or HIF1α levels, which are driven by feeding–fasting cycles or hypoxic stress [87], may similarly shift the balance of E‐box occupancy, contributing to the reprogramming of circadian gene expression (see below).

Despite increasing insight into these interactions, current technologies still fall short in providing a comprehensive picture of dynamic, locus‐specific competition among bHLH TFs *in vivo*. Most ChIP‐based methods lack the resolution to capture real‐time competitive binding events. Single‐molecule techniques such as SMF offer greater granularity, but are currently limited in their ability to distinguish among multiple co‐occupying TFs at protected motifs. Future advances in high‐throughput single‐molecule occupancy profiling will be essential for quantitatively dissecting how different bHLH TFs partition access to the genome under various cellular conditions.

## Regulation of rhythmic transcription by other circadian transcription factors—mechanistic parallels and divergences

While the mammalian TTFL is initiated by CLOCK : BMAL1, a broader network of circadian TFs reinforces the robustness of molecular oscillations and drives rhythmic expression of hundreds to thousands of downstream clock‐controlled genes. Among these, two major classes play critical roles in shaping the temporal transcriptional landscape across tissues: nuclear receptors (NRs) and PAR bZIP TFs (Fig. 1). The nuclear receptors RORα, RORβ, and RORγ function as transcriptional activators, while their antagonists REV‐ERBα (NR1D1) and REV‐ERBβ (NR1D2) act as repressors. These TFs bind to ROREs, which contain a consensus A[A/T]NTAGGTCA motif, and exert opposing regulatory effects on shared genomic targets. Similarly, the PAR bZIP transcriptional activators DBP, TEF, and HLF, as well as the repressor NFIL3 bind to D‐box elements (consensus: TTATG[T/C]AA) and contribute to the rhythmic regulation of a distinct subset of CCGs [4, 5].

A striking feature of both RORE‐ and D‐box regulated networks is that each includes TFs with opposing regulatory functions (activators and repressors) that bind the same DNA motif, in contrast to the E‐box system. As a result, ROREs and D‐boxes likely remain occupied throughout the 24‐h cycle, with binding alternately dominated by activators or repressors, in antiphase approximately 12 h apart. Although direct evidence from SMF remains limited, this model predicts persistent DNA occupancy at these elements, with temporal shifts in transcriptional output reflecting changes in TF identity rather than chromatin accessibility *per se*. Consistent with this model, nucleosome occupancy at ROREs and D‐boxes does not appear to be rhythmic, in contrast to what is observed at E‐boxes [62]. One possible explanation is that the continuous competition between DNA‐bound TFs (activators or repressors) and histones prevents cyclical nucleosome assembly. Alternatively, the chromatin remodelers recruited by the activators and repressors may be unable to create a substantially different chromatin remodeling, thereby resulting in similar chromatin accessibility across time points.

The lack of rhythmic nucleosome occupancy around ROREs and D‐boxes likely prevents nucleosome‐mediated TF cooperativity, a phenomenon proposed to stabilize cooperative TF binding through dynamic competition with nucleosomes [68]. However, this does not preclude other forms of rhythmic TF‐driven cooperativity, including temporal changes in TF availability or DNA binding affinity. In addition, rhythmic binding of RORα or DBP to DNA may facilitate the recruitment or stabilization of additional TFs at nearby motifs even in the absence of chromatin remodeling. Such cooperativity may arise through direct protein–protein interactions and/or DNA‐guided TF interactions [88]. It could also involve DNA‐mediated allostery, a mechanism where the binding of one TF induces conformational changes in the DNA that enhance the binding of another nearby TF [89]. This principle has been observed in other systems where TF pairs interact indirectly via shared DNA deformation, even in the absence of stable protein–protein contact [90, 91, 92, 93].

REV‐ERB‐mediated transcriptional repression has been strongly linked to the rhythmic recruitment of the nuclear receptor corepressor (NCoR) complex, a multiprotein assembly that includes histone deacetylase 3 (HDAC3) [94, 95]. Upon binding to ROREs, REV‐ERBs direct NCoR‐HDAC3 to target loci, leading to histone deacetylation, chromatin compaction, and suppression of transcription. This mechanism has been implicated in the rhythmic repression of key metabolic and circadian genes in the liver and other peripheral tissues. Moving forward, it will be critical to integrate SMF with histone modification mapping (e.g., detection of H3K27ac and H3K9ac) to directly test the hypothesis that REV‐ERBs' occupancy is coupled to the loss of active histone marks and the establishment of repressive chromatin states that disfavor Pol II recruitment. Such multimodal approaches could reveal not only when and where specific TFs are bound, but also how these interactions translate into dynamic chromatin landscapes and rhythmic transcriptional outputs.

## Interplay between circadian transcription factors and other transcription factors—implications for the reprogramming of cycling transcriptomes

**Fig. 3:**
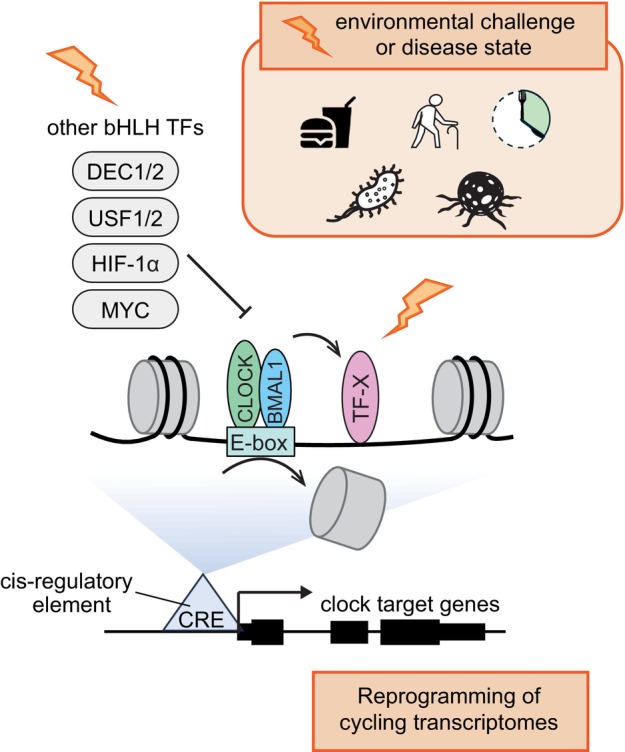
CLOCK:BMAL1 operates in a highly combinatorial and context‐dependent regulatory network. At cis‐regulatory elements (CREs) targeted by CLOCK:BMAL1, the rhythmic binding of CLOCK:BMAL1 to E‐box promotes rhythmic nucleosome removal, which can facilitate access for other TFs (e.g., TF‐X, colored in pink) in a time‐of‐day‐dependent manner. bHLH TFs other than CLOCK:BMAL1 (colored in light gray) can also recognize and bind the E‐box motif, leading to competition between CLOCK:BMAL1 and these other basic helix–loop–helix (bHLH) TFs for E‐box access. The expression and binding of these other TFs may be susceptible to environmental challenges and/or disease states, including high fat diet, aging, fasting, cancer, inflammation, hypoxia, etc. (illustrated by the orange thunderbolt). This may affect how CLOCK:BMAL1 cooperate with these other TFs, and in turn result in altered CRE transcriptional activity and reprogramming of cycling transcriptomes.

As discussed above, CLOCK:BMAL1 interplay with non‐circadian TFs involves nucleosome‐mediated assisted TF loading and competition with other bHLH TFs for E‐box access. An important consequence of this interplay is that fluctuations in the abundance, stability, or activity of these other noncircadian TFs, including after environmental challenges or in disease states, are likely to strongly impact CRE activity and, ultimately, the rhythmic transcription of nearby target genes. Importantly, alteration of target gene rhythmic transcription would occur without any strong effect on CLOCK:BMAL1 DNA binding affinity, and thus without any strong effects on the TTFL functioning (Fig. 3). Given that the interplay between CLOCK : BMAL1 and other TFs is largely CRE specific, this provides a mechanistic underpinning for how environmental challenges and disease states reprogram cycling transcriptomes, that is, how they profoundly reshape the nature of CCGs without altering core clock oscillations. Such reprogramming of rhythmic gene expression have been extensively described, including under conditions as diverse as high‐fat diet, fasting, aging, and exposure to antibiotics or lipopolysaccharides [18, 19, 20, 21, 22, 23, 24, 25]. For example, high‐fat diet increases total PPARγ protein level in mouse liver with nuclear level gaining significant oscillation, resulting in *de novo* cycling of genes that were otherwise arrhythmic, all without effecting core clock genes oscillations [18]. Additionally, time restricted feeding and fasting alters the activity of metabolic TFs including CREB and SREBP in liver, along with the rhythmic expression of their target genes [96].

It is worth highlighting that the expression of some bHLH TFs that compete with CLOCK:BMAL1 for E‐box access varies dramatically based on the cellular environment. For instance, MYC is typically expressed at low levels in most adult tissues but becomes highly activated in various cancers [97]. Similarly, HIF1α, another E‐box‐binding TF, is degraded under normoxic conditions but stabilized during hypoxia [98]. These shifts in expression can alter the stoichiometry and competition landscape at specific CLOCK : BMAL1‐bound enhancers, potentially leading to global rewiring of circadian transcriptional networks in disease states.

CLOCK:BMAL1‐mediated chromatin opening is not limited to enhancing access at the immediately nearby binding sites. The displacement of nucleosomes, which occupies a ~ 147‐bp DNA footprint, can expose adjacent motifs located tens to a couple of hundred base pairs away, enabling the binding of TFs with distinct sequence preferences. Given the strong enrichment in TF binding motifs at CREs, CLOCK:BMAL1‐mediated chromatin opening and nucleosome repositioning may affect the loading of virtually any noncircadian TFs, assuming that their binding to DNA is responsive to the chromatin remodeling. Thus, any changes in TF activity upon environmental challenge or disease state can potentially change the chromatin landscape at CLOCK:BMAL1 CREs, with ensuing impacts on target gene expression. Altogether, such mechanisms help explain how rhythmic transcription can remain plastic and responsive to environmental inputs (e.g., from nutrient state to hypoxia or oncogenic signaling) despite stable oscillations of the core clock components.

## Conclusions and perspectives

The regulation of rhythmic gene expression by CLOCK:BMAL1 is far more nuanced than previously envisioned. Rather than functioning in isolation, CLOCK:BMAL1 operates within a highly combinatorial and context‐dependent regulatory network, where chromatin architecture, TF cooperativity, and environmental inputs converge to shape CRE activity. SMF has improved our understanding of how CLOCK:BMAL1 modulates nucleosome positioning and facilitates the binding of other transcription factors. The concept of facilitated loading introduces a framework in which CLOCK:BMAL1 not only initiates transcription but also gates access for other TFs in a time‐dependent manner. This mechanism offers a plausible explanation for the extensive plasticity observed in circadian transcriptional programs across tissues and environmental conditions. Moreover, long‐range TF cooperativity, potentially involving CLOCK:BMAL1 and distant partners, suggests that circadian regulation may extend across broader regulatory domains than previously appreciated.

Extending SMF to classic circadian models such as *Drosophila melanogaster* and *Neurospora crassa* could also prove transformative. These organisms have their own circadian TFs, for example, CLK : CYC in flies and WHITE COLLAR complex in fungi, that engage regulatory DNA in ways both parallel to and distinct from CLOCK:BMAL1 [3, 99]. Because many of the mechanistic principles underlying circadian transcription, such as nucleosome remodeling, TF cooperativity, and enhancer priming, are conserved across eukaryotes, high‐resolution footprinting techniques in these models would reveal common regulatory logics as well as species‐specific adaptations. Moreover, integrating SMF data from diverse systems would provide a powerful comparative framework to test whether features such as facilitated loading and long‐range cooperativity are universal strategies for circadian transcription or represent lineage‐specific innovations.

Several open questions remain. How do chromatin modifications and histone variants intersect with CLOCK:BMAL1 dynamics? Can rhythmic chromatin priming be used to build predictive models to anticipate transcriptional outputs? Future work should aim to combine high‐resolution SMF with antibody‐based sequencing approaches, live‐cell imaging, perturbation assays, and multi‐omic integration to dissect the temporal logic of CLOCK:BMAL1‐bound CREs *in vivo*. Together, these findings redefine CLOCK:BMAL1 not just as a circadian activator, but as a central organizer of enhancer architecture and transcriptional timing, thus establishing a conceptual bridge between circadian biology, epigenomics and gene regulatory networks.

## Author contributions

XYN and JSM drafted and wrote the paper.
